# Comparative genome sequence analysis of several species in the genus *Tepidimonas* and the description of a novel species *Tepidimonas charontis* sp. nov.

**DOI:** 10.1099/ijsem.0.003942

**Published:** 2020-01-02

**Authors:** Luciana Albuquerque, Nadine Castelhano, Pedro Raposo, Hugo J. C. Froufe, Igor Tiago, Rita Severino, Inês Roxo, Inês Gregório, Cristina Barroso, Conceição Egas, Milton S. da Costa

**Affiliations:** ^1^​ Center for Neuroscience and Cell Biology, University of Coimbra, 3004-504 Coimbra, Portugal; ^2^​ Next Generation Sequencing Unit, Biocant, BiocantPark, Núcleo 04, Lote 8, 3060-197 Cantanhede, Portugal; ^3^​ Center for Functional Ecology, University of Coimbra, 3000-456, Coimbra, Portugal

**Keywords:** Genome, New taxa, *Betaproteobacteria*, *Tepidimonas charontis* sp. nov.

## Abstract

We performed high-quality genome sequencing of eight strains of the species of the genus *Tepidimonas* and examined the genomes of closely related strains from the databases to understand why *Tepidimonas taiwanensis* is the only strain of this genus that utilizes glucose and fructose for growth. We found that the assimilation of these hexoses by *T. taiwanensis* was due to the presence of two transporters that are absent in all other genomes of strains of members of the genus *Tepidimonas* examined. Some strains lack genes coding for glucokinase, but the Embden–Meyerhof–Parnas pathway appears to be otherwise complete. The pentose phosphate pathway has a complete set of genes, but genes of the Entner–Doudoroff pathway were not identified in the genomes of any of the strains examined. Genome analysis using average nucleotide identity (ANIb), digital DNA–DNA hybridization (dDDH), average amino acid identity (AAI) and phylogenetic analysis of 400 conserved genes was performed to assess the taxonomic classification of the organisms. Two isolates of the genus *Tepidimonas* from the hot spring at São Pedro do Sul, Portugal, designated SPSP-6^T^ and SPSPC-18 were also examined in this study. These organisms are mixotrophic, have an optimum growth temperature of about 50 ºC, utilize several organic acids and amino acids for growth but do not grow on sugars. Distinctive phenotypic, 16S rRNA gene sequence and genomic characteristics of strains SPSP-6^T^ and SPSPC-18 lead us to propose a novel species based on strain SPSP-6^T^ for which we recommend the name *Tepidimonas charontis* sp. nov. (=CECT 9683^T^=LMG 30884^T^).

The slightly thermophilic species of the genus *Tepidimonas* are classified in the class *Betaproteobacteria* and the order *Burkholderiales *, but this genus is not assigned to a family by LPSN (http://www.bacterio.net/-classifphyla.html#tepidimonas). The genus comprises seven species with validly published names, *Tepidimonas ignava* [1], *Tepidimonas aquatica* [2], *Tepidimonas taiwanensis* [3], *Tepidimonas thermarum* [4] and *Tepidimonas fonticaldi* [5], *Tepidimonas sediminis* and *Tepidimonas alkaliphilus* [6], while the species ‘*Tepidimonas arfidensis*’ [7] has not been validly published.

With the exception of *T. taiwanensis *, none of the strains of this genus grow in a minimal medium with glucose and fructose as sole carbon and energy source. The type strains of all species of this genus assimilate individual organic acids and amino acids for growth. Moreover, the strains examined oxidize thiosulfate in the presence of an organic carbon source, indicating that the strains are mixotrophic.

Chemoorganotrophic and mixotrophic bacteria that do not utilize sugars for growth are not rare; these organisms may lack specific sugar transporters or enzymes involved in the Embden–Meyerhof–Parnas, the Entner–Doudoroff or the pentose phosphate pathways. With the objective of understanding the conundrum that only one type strain of this genus is capable of growing on hexoses, we performed high-quality draft genome sequencing of eight type strains of *T. ignava *, *T. aquatica *, *T. fonticaldi *, *T. taiwanensis *, *T. thermarum *, *T. sediminis *, *T. alkaliphilus* and the type strain of one isolate of the genus *Tepidimonas* from the hot spring at São Pedro do Sul, Portugal, designated SPSP-6^T^. We also scrutinized two genome sequences of strains closely related to the type strain of *T. taiwanensis* I1-1^T^, namely strains VT154-175 and MB2 as well as a strain closely related to *T. fonticaldi* designated PL17 [8, 9]. We also propose that strain SPSP-6^T^ represents a novel species for which we recommend the name *Tepidimonas charontis* sp. nov.

Strains SPSP-6^T^ and SPSPC-18 were isolated from water samples at the hot spring at São Pedro do Sul in Central Portugal (40° 46′ N, 8° 4′ W) with temperatures of 65 and 50 °C, respectively. The samples were maintained without temperature control for 1 day. Samples or dilutions of the water samples were filtered through membrane filters (Gelman type GN-6; pore size 0.45 µm; diameter 47 mm). The filters were placed on the surface of solidified *Thermus* medium (https://www.dsmz.de/microorganisms/medium/pdf/DSMZ_Medium1033.pdf) [10]. The plates were wrapped in plastic to prevent evaporation and incubated at 50 °C for up to 5 days. Cultures were purified by sub-culturing and the isolates stored at –70 °C in *Thermus* medium with 15 % (w/v) glycerol. Cultivation in Degryse medium 162 [11], containing 0.25 % yeast extract and 0.25 % tryptone, was later adopted because this medium resulted in higher growth yields. The strains of *T. alkaliphilus* YIM 72238^T^ (KCTC 52717^T^), *T. aquatica* CLN-1^T^ (DSM 14833^T^), *T. fonticaldi* AT-A2^T^ (KCTC 23862^T^), *T. ignava* SPS-1037^T^ (DSM 12034^T^), *T. sediminis* YIM 72259^T^ (NBRC 112410^T^), *T. taiwanensis* I1-1^T^ (LMG 22826^T^) and *T. thermarum* AA-1^T^ (LMG 23094^T^) were used for comparative purposes.

The growth temperature ranges of SPSP-6^T^ and SPSPC-18 were examined with 5 °C increments between 20 and 65 °C by measuring the turbidity at 610 nm of cultures in liquid Degryse medium as described previously. The pH range for growth was examined at 50 °C in the same medium by using 50 mM MES, HEPES, TAPS and CAPSO over a pH range of 6.0 to 10.0 with 0.5 unit increments, in a rotary water-bath shaker. Growth with added salt, 0.5 and 1 % (w/v) NaCl, was determined in liquid medium. Catalase and oxidase activities and nitrate reduction were examined as described previously [12].

Single-carbon source assimilation tests were performed in a minimal medium composed of Degryse medium 162 basal salts containing filter-sterilized single carbon sources (2.0 g l^−1^), ammonium sulfate (0.5 g l^−1^) and a vitamin and nucleotide solution at a final concentration of 40 µg l^−1^ [4]. Growth of the strains on single carbon sources was examined by measuring the turbidity of cultures (at 610 nm) in 20 ml screw-capped tubes containing 10 ml medium for up to 7 days.

Growth on thiosulfate was assessed on modified 69 medium (https://www.dsmz.de/microorganisms/medium/pdf/DSMZ_Medium69.pdf) containing the following components per litre: Na_2_HPO_4_.12H_2_O, 10.6 g; KH_2_PO_4_, 1.5 g; NH_4_Cl, 0,3 g; yeast extract, 1.0 g; 1 ml trace elements solution SL-6 of medium 27 (https://www.dsmz.de/microorganisms/medium/pdf/DSMZ_Medium27.pdf) without the addition of sulfate. A vitamin and nucleotide solution consisting of thiamine, riboflavin, pyridoxine, biotin, folic acid, inositol, nicotinic acid, pantothenic acid, *p*-aminobenzoic acid, cyanocobalamin, adenine, thymine, cytosine, guanine, cytidine, uracil and inosine was added to modified 69 medium at a final concentration of 40 µg l^−1^ [4]. Concentrations of 0.5 and 1 g l^−1^ of thiosulfate was added to this media. At appropriate intervals, the turbidity of the cultures was measured and the levels of thiosulfate and sulfate in the supernatants were measured using the methods described by Sörbo 1987 [13] and Westley 1987 [14]. Motility of SPSP-6^T^ and SPSPC-18 was examined by phase-contrast microscopy (1000×) and with the Ryu strain [15].

Cultures for fatty acid analysis were grown on R2A and Degryse medium 162 at 50 °C for 24 h. Fatty acid methyl esters (FAMEs) were obtained from fresh wet biomass, separated, identified and quantified with the standard MIS Library Generation Software, version 6.0, aerobe TSBA method (Microbial ID, MIDI) as described previously [16].

Total genomic DNA of *T. thermarum* AA-1^T^ (LMG 23094^T^), *T. ignava* SPS-1037^T^ (DSM 12034^T^), *T. aquatica* CLN-1^T^ (DSM 14833^T^), *T. fonticaldi* AT-A2^T^ (KCTC 23862^T^), *T. taiwanensis* I1-1^T^ (LMG 22826^T^), *T. sediminis* YIM 72259^T^ (NBRC 112410^T^), *T. alkaliphilus* YIM 72238^T^ (KCTC 52717^T^) and SPSP-6^T^ (LMG 30884^T^) was extracted following the method of Nielsen *et al*. [17]. The purity of the DNA was verified by 1 % agarose gel electrophoresis. DNA quantity was measured by fluorescence in an Invitrogen Qubit 2.0 fluorometer (Thermo Fisher Scientific). The DNA was prepared for genome sequencing using the Nextera XT DNA Library Preparation Kit (Illumina). Bacterial genomes were sequenced on the MiSeq (Illumina) with paired-end (PE) 2×300 bp reads. The draft genomes of strains MB2 (GCF_001481285.1), VT154-175 (GCF_000807215.1) and PL17 (GCF_001675355.1) of members of the genus *Tepidimonas* were obtained from public databases.

Sequenced reads were filtered for quality with Trimmomatic (version 0.30 [18]; and assembled with SPAdes (version 3.9.1 [19]). The resulting contigs were annotated with Prokaryotic Genome Prediction 2 (PGP2). PGP2 uses Prodigal (version 2.6 [20]) for gene prediction, Barrnap (version 0.8; https://github.com/tseemann/barrnap) for rRNA and tRNA genes detection, and Prokka (version 1.12 [21]) for the annotation of protein-coding genes. Gene annotation with Prokka uses the SwissProt [22], HAMAP [23], TIGRFAMs [24] and Pfam [25] repositories. Genes observed to be missing in the pathways were searched manually at the ends of the contigs and were annotated. These genes were the sulfur-oxidizing protein *soxZ* of *T. aquatica *, strain SPSP-6^T^, *T. taiwanensis* I1-1^T^ and strain MB2, the phosphoglycerate kinase gene (*pgk*) of strain SPSP-6^T^ and the alpha chain of the nitrate reductase *narG* of *T. thermarum *. Genome estimated completeness and contamination were verified with CheckM (version 1.0.7) [26].

Multiple sequence alignments were performed using muscle [27]. Phylogenetic trees were reconstructed with the neighbor-joining (NJ) and maximum-likelihood (ML) algorithms using mega (version X) [28]. For the NJ and ML algorithms, genetic distances were calculated with the Jukes–Cantor model [29]. Bootstrap analysis based on 1000 replicates was used to evaluate the resulting tree topologies.

Pairwise average nucleotide identity based on blast (ANIb) was analyzed with JSpecies [30]. Digital DNA–DNA hybridization (dDDH) was determined with the Genome-to-Genome distance Calculator [31]. The amino acid identity (AAI) phylogenetic tree based on 400 universally conserved protein sequences was produced with PhyloPhlAn [32] to provide additional information on the relationships between members of the genus *Tepidimonas *.

The assembled genomes of the strains of members of the genus *Tepidimonas* ranged from 2465 kbp for *T. alkaliphilus* strain YIM 72238^T^ to 3009 kbp for strain *T. fonticaldi* AT-A2^T^ (Table 1). The DNA G+C content of genomes ranged from 66.63 % for strain SPSP-6^T^ to 71.83 % for *T. sediminis* YIM 72259^T^. The completeness of the genomes examined ranged from 98.91 % for the draft genomes of strains VT154-175 and AA-1^T^ to 100 % for the draft genome of *T. fonticaldi* AT-A2^T^. The genomes of strains of members of the genus *Tepidimonas* had a variable number of rRNA genes ranging from three in *T. alkaliphilus* YIM 72238^T^ to twelve in the genome of strain VT154-175.

**Table 1.** Summary of genome sequencing and annotation metrics Strains: 1, *Tepidimonas alkaliphilus* YIM 72238^T^ (VJNB00000000); 2, *Tepidimonas aquatica* CLN-1^T^ (VJNA00000000); 3, *Tepidimonas fonticaldi* AT-A2^T^ (VJOO00000000); 4, PL17 (GCF_001675355.1); 5, *Tepidimonas ignava* SPS-1037^T^ (VJNC00000000); 6, *Tepidimonas sediminis* YIM 72259^T^ (VJND00000000); 7, SPSP-6^T^ (VJON00000000); 8, *Tepidimonas taiwanensis* I1-1^T^ (VJOM00000000); 9, MB2 (GCF_001481285.1); 10, VT154-175 (GCF_000807215.1); 11, *Tepidimonas thermarum* AA-1^T^ (VJOL00000000).

	1	2	3	4	5	6	7	8	9	10	11
Assembled genome size (bp)	2 465 445	2 672 904	3 009 257	2 740 548	2 715 700	2 533 936	2 808 982	2 859 782	2 813 615	2 924 885	2 703 753
DNA G+C content (%)	69.01	68.55	69.00	69.53	68.79	71.83	66.63	68.80	68.80	68.66	68.70
Protein-coding genes	2280	2507	2758	2519	2563	2337	2634	2622	2591	2658	2552
Genes with function prediction	2049	2256	2407	2290	2217	2141	2208	2310	2291	2362	2260
Ribosomal genes (5S, 16S, 23S)	1, 1, 1	2, 2, 2	2, 2, 2	2, 1, 1	2, 2, 2	2, 1, 1	2, 2, 2	2, 2, 2	2, 2, 2	4, 4, 4	2, 2, 2
Estimated genome completeness (%)	99.51	99.53	100	99.53	99.53	99.07	99.14	99.42	99.42	98.91	98.91
Estimated contamination (%)	0.00	0.96	0.00	1.17	0.03	0.15	1.05	0.03	0.18	0.05	0.47

Genes coding for enzymes involved in the hydrolysis of starch, cyclodextrin and pullulan, namely alpha-amylase (EC 3.2.1.1), beta-amylase (EC 3.2.1.2), pullulanase (EC 3.2.1.41) and cyclomaltodextrinase (EC 2.4.1.19), were not identified in the genomes of any strains of members of the genus *Tepidimonas *. Therefore, it should not be possible for these organisms to obtain glucose or maltose from starch that could be taken up in pure culture. The type strain of *T. taiwanensis* has been reported to hydrolyze starch [3], while another study has reported that starch was not hydrolyzed by the same organism, corroborating the absence of starch hydrolyzing-enzymes from the genome analyses [4].

The ability of the type strain of *T. taiwanensis* to grow on glucose and fructose has been reproduced in laboratories that examined these phenotypic characteristics [4, 6]. Likewise, the inability of the other species of this genus to grow on hexoses is also well attested. The genome sequences of the organisms used in this study clarified the likely reasons why the type strain of *T. taiwanensis* is able to use glucose and fructose while the other strains are not (Table S1, available in the online version of this article). The genome analysis indicated that glucose and fructose transporters only occur in the type strain of *T. taiwanensis *, as well as strains MB2 and VT154-175, where putative ABC glucose/mannose (*gtsABCD*) and fructose (*frcABC*) transporters were the only two sugar transporters identified. Moreover, we did not identify other transport systems for hexoses, disaccharides or pentoses in the genomes of any of the strains of members of the genus *Tepidimonas *. The gene coding for glucokinase (EC 2.7.1.2) was only identified in the genomes of *T. aquatica* CLN-1^T^, *T. taiwanensis* I1-1^T^, strains MB2 and VT154-175. Otherwise all other genes of the Emden–Meyerhof–Parnas pathway were identified in the genomes of strains of members of the genus *Tepidimonas *.

The genes coding for the enzymes of the pentose phosphate pathway, specifically glucose-6-phosphate 1-dehydrogenase (EC 1.1.1.49), 6-phosphogluconolactonase (EC 3.1.1.31), 2-dehydro-3-deoxyphosphogluconate aldolase (EC 4.1.2.14) and phosphogluconate dehydratase (EC 4.2.1.12), were also identified in the genomes of *T. taiwanensis* I1-1^T^, strains MB2 and VT154-175 but were not identified in any of the other genomes. The pentose phosphate pathway can be predicted to channel intermediates to glyceraldehyde-3-phosphate. Additionally, the gene coding for the enzyme 6-phosphogluconate dehydrogenase (EC 1.1.1.44, EC 1.1.1.343) was not identified in any of the genomes analyzed, thus precluding the utilization of the Entner–Doudoroff pathway by all strains. Gluconeogenesis, as expected, was predicted in all strains of members of the genus *Tepidimonas* examined because the key enzyme fructose-1,6-bisphosphatase (EC 3.1.3.11) was identified in all genomes.

Although the type strain of *T. taiwanensis* grows on glucose and fructose, this strain, like all strains of this genus, does not grow on any other carbohydrates examined, such as mannose, galactose, trehalose, maltose, sucrose, ribose, l-arabinose, xylose or polyol (Table S1). We were unable to identify genes in any strains of members of the genus *Tepidimonas* that could channel these carbohydrates to the Emden–MeyerhofParnas or the pentose phosphate pathways.

Enzymes of the TCA cycle were identified in all genomes of members of the genus *Tepidimonas *. Genes coding for the enzymes of oxidative phosphorylation were NADH dehydrogenase (EC 1.6.5.11, complex I), succinate dehydrogenase/fumarate reductase (EC 1.3.5.1, complex II), cytochrome bc1 (EC 1.10.2.2, complex III), cytochrome c oxidase cbb3-type (EC 1.9.3.1, complex IV) and an F-type ATPase (EC 3.6.3.14, complex V) in all genomes.

Experimental nitrate reduction to nitrite has been observed for the type strains of *T. aquatica *, *T. fonticaldi *, *T. thermarum and T. taiwanensis* but not or the type strain of *T. ignava* and strain SPSP-6^T^ [5]. The strains of the members of the genus *Tepidimonas* have variable genes involved in nitrogen metabolism (Table S2). For example, the type strain of *T. fonticaldi* possesses the most complete set of genes of the species of this genus, being predicted to be capable of reducing nitrate to nitrous oxide via the products of *norB* (nitric oxide reductase, large subunit) and *norC* (nitric oxide reductase, small subunit). The other strains, including the closely related strain PL17, appear to lack genes *norB* and *norC*. Genes coding for nitrate/nitrite transporters *nasA/narK* and the nitrate reductase complex *narGHIJ* were identified in the genomes of *T. aquatica* CLN-1^T^, *T. fonticaldi* AT-A2^T^, strain PL17, *T. taiwanensis* I1-1^T^, strain VT154-175 and *T. thermarum* AA-1^T^ *,* but not in *T. alkaliphilus* YIM 72238^T^, strain SPSP-6^T^ and strain MB2. *T. ignava* SPS-1037^T^ and *T. sediminis* YIM 72259^T^ have genes coding for the nitrate/nitrite transporters *nasA/narK* but the nitrate reductase complex *narGHIJ* was not identified in the genome sequences. Nitrate did not appear to be reduced by *T. thermarum* AA-1^T^ experimentally in one study but his strain has been reported to reduce nitrate in another study [4, 5]. However, the genome predicts that nitrate should be reduced to nitrite because this organism possesses *narGHIJ*. The only gene involved in the reduction of nitrate identified in SPSP-6^T^ and *T. alkaliphilus* YIM 72238^T^ was *NirB* (nitrite reductase, NADH-dependent large subunit) (Tables S1 and S2).

With the exception of strains PL17, MB2 and VT154-175 whose phenotypic characteristics are not available, and the type strains of *T. sediminis* and *T. alkaliphilus* where thiosulfate oxidation was not examined, all other strains of members of the genus *Tepidimonas* oxidize thiosulfate to sulfate experimentally. However, all genomes predict that thiosulfate is oxidized to sulfate via the *sox* pathway, namely the *soxXABCDYZ* genes.

The three subunit orthologs of the tripartite ATP-independent periplasmic transporter (TRAP) that transport the C_4_-dicarboxylates malate/fumarate (DctM, DctP and DctQ) were identified in *T. aquatica* CLN-1^T^, *T. ignava* SPS-1037^T^, *T. alkaliphilus* YIM 72238^T^ and *T. taiwanensis* I1-1^T^. These organisms use malate and fumarate as single carbon sources [33]. The other type strains appear to have only the genes for the DctM and DctP components or the DctM component alone. The type strain of *T. thermarum* and strain SPSP-6^T^ do not grow on malate or fumarate. The type strain of *T. fonticaldi* does not grow on malate but growth on fumarate was not tested, while the type strains of *T. sediminis* and *T *. *alkaliphilus* were not tested for the utilization of malate or fumarate (Table S1).

We only identified the genes for the tripartate tricarboxylate ABC system transporter for citrate composed of three subunits (TctA, TctB and TctC) in the genome of the type strain of *T. taiwanensis* and strain MB2. Strain VT154-175 has two components (TctB and TctC), while the other strains of members of the genus *Tepidimonas* appear to have only one component [34]. The type strain of *T. taiwanensis* is the only organism, among those examined that uses citrate for growth (Table S1).

The pairwise 16S rRNA gene sequence similarity determined between strains SPSP-6^T^ and SPSC-18 was 100 %. These isolates shared pairwise 16S rRNA gene sequence similarities of 98.07, 98.38 and 98.44 % with the type strains of *T. ignava *, *T. aquatica* and *T. taiwanensis *. (Fig. 1, Table S3). The sequence similarity between *T. fonticaldi* AT-A2^T^ and strain PL17 was 99.86 %, indicating an extremely close relationship between the two organisms. A close relationship of *T. fonticaldi* AT-A2^T^ with ‘*T. arfidensis*’ of 99.58 % was also noted. The 16S rRNA gene sequence analysis also indicated *T. taiwanensis* I1-1^T^ to be closely related to strains MB2 and VT154-175 with sequence similarities of 99.93 and 99.58 %, respectively (Table S3). The phylogenetic results of 16S rRNA gene analysis indicate that SPSP-6^T^ is located within a cluster comprising the type strains of *T. ignava *, *T. aquatica *, *T. taiwanensis *, *T. alkaliphilus* and *T. sediminis *. However, SPSP-6^T^ is most closely related to the type strains of *T. ignava *, *T. aquatica* and *T. taiwanensis* (Figs 1 and S1). The phylogenetic results based on 400 conserved genes were consistent with the 16S rRNA gene sequence results (Fig. 2), corroborating the phylogenetic relations observed for SPSP-6^T^ within the genus *Tepidimonas *. The phylogenetic analysis of 400 conserved genes also showed *T. taiwanensis* I1-1^T^, strains MB2 and VT154-175 to be very closely related to each other indicating that the three strains represent one species. Strain PL17 and *T. fonticaldi* AT-A2^T^ should also probably be regarded as representing one species.

**Fig. 1.**
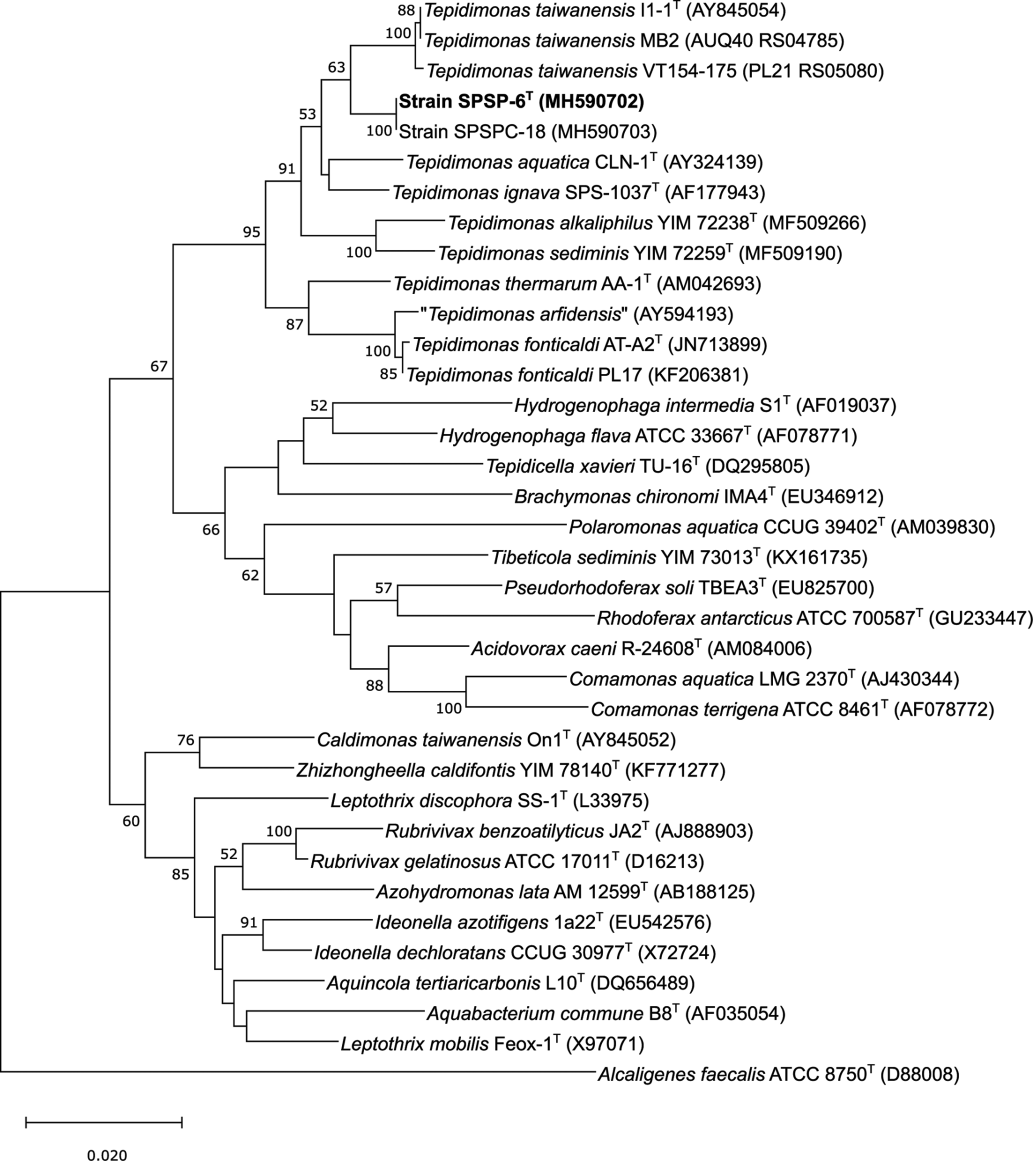
Phylogenetic reconstruction based on 16S rRNA genes of strains of members of the genus *Tepidimonas* using the neighbor-joining algorithm. The numbers at branching points represent bootstrap values from 1000 replications. Bar, 0.02 substitutions per nucleotide position. The tree was rooted using the sequence of *Alcaligenes faecalis* ATCC 8750^T^ (D88008).

**Fig. 2.**
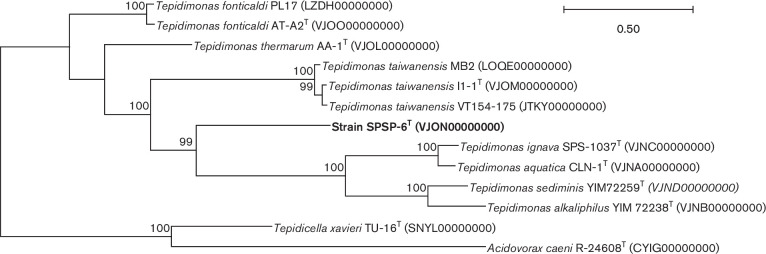
Phylogenetic tree reconstruction of members of the genus *Tepidimonas* based on a set of 400 conserved bacterial genes. GenBank accession numbers of the genomes are given in parentheses. Bootstrap values were calculated based on 1000 replicates. The scale bar indicates the number of amino acid substitutions per site.

Additionally, the results of the genome comparisons, namely ANIb, AAI and dDDH corroborated the results of the 16S rRNA sequence analysis regarding the distinct species nature of the lineages (Tables 2, S4 and S5). Based on a threshold value of 95–96 % for species delineation [35, 36], the ANIb values indicate that SPSP-6^T^, with ANIb values of about 80 % with other species constitutes a separate species of the genus *Tepidimonas *. The ANIb value for *T. fonticaldi* AT-A2^T^ and strain PL17 of 97.46 % indicates a very close relationship between these strains. The same is true of *T. taiwanensis* I1-1^T^ with strains MB2 and VT154-175 that share ANIb values of 97.53 to 98.48 %. In addition to the genomic values, these are the only currently known strains that have genes for the transport of glucose and fructose, and utilize these hexoses for growth. On the basis of the ANIb values, the type strains of *T. aquatica* and *T. ignava* are closely related (93.22 %). Nevertheless, this value is below the threshold value for delineation of species (Table 2).

**Table 2.** Average nucleotide identity (ANIb, %) values between genomes of members of the genus *Tepidimonas* and strains of *Acidovorax caeni* and *Tepidicella xavieri
* The percentages of aligned nucleotides are given in brackets. Strains: 1, *Tepidimonas alkaliphilus* YIM 72238^T^ (VJNB00000000); 2, *Tepidimonas aquatica* CLN-1^T^ (VJNA00000000); 3, *Tepidimonas fonticaldi* AT-A2^T^ (VJOO00000000); 4, PL17 (GCF_001675355.1); 5, *Tepidimonas ignava* SPS-1037^T^ (VJNC00000000); 6, *Tepidimonas sediminis* YIM 72259^T^ (VJND00000000); 7, SPSP-6^T^ (VJON00000000); 8, *Tepidimonas taiwanensis* I1-1^T^ (VJOM00000000); 9,MB2 (GCF_001481285.1); 10, VT154-175 (GCF_000807215.1); 11, *Tepidimonas thermarum* AA-1^T^ (VJOL00000000); 12, *Acidovorax caeni* R-24608^T^ (GCF_001298675.1); 13, *Tepidicella xavieri* TU-16^T^ (GCF_004363315.1)

	1	2	3	4	5	6	7	8	9	10	11	12	13
**1**	–	81.13 [68.68]	81.09 [63.44]	80.88 [65.69]	80.90 [68.25]	87.96 [75.53]	77.99 [55.16]	78.87 [60.48]	79.12 [59.76]	78.86 [60.54]	79.59 [63.23]	72.89 [34.46]	74.86 [48.8]
**2**	81.01 [63.78]	–	81.57 [67.81]	81.36 [66.74]	93.27 [79.68]	82.37 [69.13]	78.80 [54.28]	79.45 [60.25]	80.06 [60.00]	79.50 [60.25]	79.65 [66.93]	72.85 [34.36]	75.80 [51.47]
**3**	80.88 [52.74]	81.46 [60.00]	–	97.46 [79.29]	80.16 [58.86]	82.52 [58.84]	80.57 [51.89]	81.55 [58.56]	81.95 [59.31]	81.53 [59.08]	82.67 [64.58]	73.81 [34.22]	77.07 [49.37]
**4**	80.68 [59.59]	81.29 [64.90]	97.61 [86.05]	–	80.08 [63.40]	82.51 [66.27]	80.05 [56.05]	81.45 [64.28]	81.9 [63.72]	81.48 [64.56]	82.91 [71.07]	73.72 [35.81]	76.56 [54.71]
**5**	80.89 [62.39]	93.22 [78.48]	80.00 [65.17]	80.06 [64.31]	–	81.97 [71.32]	79.58 [57.82]	79.74 [58.63]	79.88 [58.09]	79.53 [58.38]	79.84 [66.53]	72.95 [32.68]	75.36 [50.14]
**6**	87.83 [74.00]	82.26 [73.04]	82.51 [70.02]	82.63 [71.86]	81.92 [76.02]	–	79.12 [57.58]	80.42 [63.22]	80.63 [63.02]	80.43 [63.52]	80.75 [68.69]	73.33 [36.04]	75.30 [53.97]
**7**	77.99 [47.98]	78.82 [50.92]	80.65 [54.62]	80.15 [54.11]	79.84 [54.32]	79.17 [51.41]	–	79.18 [53.49]	79.43 [52.57]	79.07 [52.92]	79.42 [55.36]	72.94 [28.66]	75.11 [40.96]
**8**	78.97 [52.41]	79.48 [57.09]	81.55 [61.86]	81.45 [62.56]	79.86 [55.83]	80.56 [56.23]	79.31 [52.32]	–	97.53 [85.43]	98.48 [94.19]	80.87 [59.48]	73.27 [31.42]	75.56 [45.37]
**9**	79.10 [52.97]	79.97 [57.61]	81.86 [63.76]	81.69 [62.91]	79.94 [56.56]	80.73 [54.44]	79.24 [53.55]	97.31 [86.32]	–	96.87 [85.92]	80.93 [60.04]	73.52 [30.95]	75.87 [47.01]
**10**	78.87 [52.21]	79.64 [56.28]	81.73 [61.7]	81.46 [61.98]	79.73 [54.03]	80.68 [54.95]	79.09 [52.26]	98.48 [93.28]	97.10 [84.11]	–	80.89 [58.67]	73.52 [30.95]	75.69 [45.59]
**11**	79.50 [58.72]	79.83 [65.54]	82.77 [72.32]	82.98 [72.59]	79.96 [66.69]	80.86 [65.24]	79.36 [58.66]	81.02 [62.51]	81.27 [61.46]	81.00 [62.64]	–	73.36 [34.2]	75.81 [51.2]
**12**	72.57 [21.26]	72.70 [22.4]	73.63 [24.63]	73.63 [23.89]	72.68 [22.35]	73.23 [22.48]	72.60 [20.31]	73.13 [22.14]	73.24 [21.93]	73.22 [22.35]	73.28 [22.49]	–	73.63 [23.21]
**13**	74.80 [43.63]	75.72 [48.51]	77.11 [52.64]	76.74 [53.21]	75.31 [48.8]	75.53 [48.8]	75.04 [41.69]	75.56 [47.02]	76.16 [47.39]	75.57 [46.85]	75.79 [49.44]	73.44 [34.43]	–

The AAI values, generally taken to have a cut off value of around 70 % to delineate genera [37], indicate that the strains of members of the genus *Tepidimonas *, including strain SPSP-6^T^, belong to one genus because of higher AAI values (Table S4). The genome of *Tepidicella xavieri* has high AAI values with the species of the genus *Tepidimonas* (67.59 to 70.85 %) indicating that the organisms of the two genera are related, but within a transitional zone of AAI values, making it difficult to have an opinion on the classification of the sole strain of *Tepidicella xavieri* from the genomic analysis. However, the phylogeny obtained for the 16S rRNA gene and for 400 conserved genes sequence analysis indicates that *Tepidicella xavieri* is not closely related to the species of the genus *Tepidimonas* [38, 39].

The genomic-based dDDH estimates have values of 79.8 to 87.20 % between *T. taiwanensis* strains I1-1^T^, strains MB2 and VT154-175 (Table S5). These results are above the reference dDDH value of about 70 % to delineate separate species proposed by Stackebrandt *et al*. [35] leading us to the opinion that the three strains represent members of the species *T. taiwanensis *. Moreover, these three strains possess an ABC glucose/mannose transporter that all other strains seem to lack. The high dDDH value of 80.1 % between *T. fonticaldi* AT-A2^T^ and strain PL17 also supports the view that these two strains represent the same species. The low dDDH values between other organisms of the genus *Tepidimonas *, notably strain SPSP-6^T^ sharing no more than 25.1 %, support the view that the organisms examined represent distinct species of the genus *Tepidimonas* (Table S5).

The 16S rRNA gene sequence analysis, as well as the genomic data, circumscribes all type strains of species of the genus *Tepidimonas *, as well as strains PL17, MB2 and VT154-175 to the genus *Tepidimonas *. Moreover, strains MB2 and VT154-175 appear, based on the presence of glucose/mannose and fructose transporters and the genomic results, to represent *Tepidmonas taiwanensis*, while the close phylogenic and genomic results ascribe strain PL7 to the species *Tepidimonas fonticaldi *.

A small number of phenotypic characteristics of the type strains of the species of the genus *Tepidimonas* distinguish the strains from each other (Table S1). The fatty acid compositions of the strains were obtained after the organisms were grown in R2A and Degryse medium 162 agar plates for 24 h at 50 °C. These results indicated that the medium influenced the fatty acid composition to a large extent (Tables S6 and S7). For example, C_17 : 0_ cyclo was not detected in *T. ignava* SPS-1037^T^ and *T. sediminis* YIM 72259^T^ grown on Degryse medium 162 but reached levels of 6.7 and 7.3 %, respectively, when they were grown on R2A agar. Nevertheless, the major fatty acids of all strains were C_16 : 0_, summed feature 3 (probably C_16 : 1_ω6*c* and/or C_16 : 1_ω7*c*) and in some cases, C_17 : 0_ cyclo and summed feature 8 (C_18 : 1_ω6*c* and/or C_18 : 1_ω6*c*). However, there were differences in the concentrations of these fatty acids among the type strains. For example, the combination of C_17 : 0_ cyclo and C_17 : 0_, after growth of the organisms on R2A and Degryse medium 162, can distinguish strains SPSP-6^T^ and SPSPC-18 from the other strains of species of this genus.

Many of the validly described prokaryotic species are only based on a few distinctive phenotypic characteristics that could represent interspecific diversity, since these novel organisms are proposed on the basis of the description of one strain alone. The species of the genus *Tepidimonas* are an example of these considerations since strains SPSP-6^T^ and SPSPC-18 have identical 16S rRNA sequences but have slightly different phenotypic and fatty acid characteristics.

The novel species of the genus *Tepidimonas* represented by strain SPSP-6^T^ has very few phenotypic and chemotaxonomic characteristics that distinguish this strain from the type strains of the other species. The single carbon source assimilations, with the exception of *T. taiwanensis *, sulfur oxidation and the fatty acid composition are similar in all type strains of species of the genus *Tepidimonas* (Tables S1, S6 and S7). However, some phenotypic and chemotaxonomic characteristics indicate that the organism represents a novel species; the fatty acid composition indicated that SPSP-6^T^ and SPSPC-18 can be distinguished from other strains of members of the genus *Tepidimonas* by combining the relative proportions of C_17 : 0_ cyclo and C_17 : 0_. Except for strains SPSP-6^T^ and *T. alkaliphilus* YIM 72238^T^, which possess only homologues for *nirB*, all other strains possess genes coding for proteins involved in the reduction of nitrate to nitrite. Considering the phenotypic, genomic and the phylogenetic analysis based on 16S rRNA gene sequence and on 400 conserved genes sequences clearly confirms that strain SPSP-6^T^ represents a species level taxon, leading us to propose the name *Tepidimonas charontis* sp. nov.

## Description of *Tepidimonas charontis* sp. nov.

*Tepidimonas charontis* (cha.ron'tis. L. gen. n. *charontis* of Charon, the boatman who required payment to ferry the ancient dead Greeks across the Rivers Styx and Acheron to Hades.

Forms short rod-shaped cells 0.5–0.8 µm in width and 1.0–2.0 µm in length. Endospores are not formed. The cells Gram-stain-negative and are motile by means of one polar flagellum. Colonies on Degryse medium 162 are not pigmented and are 1 to 2 mm in diameter after 48 h of growth. The optimum growth temperature is about 50 °C; growth occurs in the range of 25–60 °C. The optimum pH is between 7.5 and 9.0; the pH range for growth is pH 6.5–9.5. Mixotrophic. Aerobic. Nitrate is not reduced to nitrite. Oxidase- and catalase-positive. The major fatty acids are C_16 : 0_ and C_16 : 1_ ω6*c* and/or C_16 : 1_ ω7*c*. Yeast extract or growth factors are required for growth. Thiosulfate is oxidized to sulfate. Several organic acids and amino acids are utilized for growth, namely succinate, lactate, pyruvate, acetate, glutamate, aspartate, l-alanine, l-asparagine, l-lysine, l-glutamine, l-isoleucine and l-ornithine, but the strains do not utilize carbohydrates or polyols.

The type strain SPSP-6^T^ (=CECT 9683=LMG 30884) was isolated from a hot spring at São Pedro do Sul in Central Portugal. The genomic DNA G+C content of the type strain is 66.63 % (determined by genome sequencing). Strain SPSPC-18 (=CECT 9684=LMG 30885) is an additional strain of this species.

GenBank/EMBL/DDBJ accession numbers for the 16S rRNA gene sequence of strains SPSP-6^T^ and SPSPC-18 are MH590702 and MH590703, respectively. The draft genome sequence of SPSP-6^T^ (VJON00000000) has been deposited in GenBank/EMBL/DDBJ.

## Supplementary Data

Supplementary material 1
