# Corrigendum: Comparative genome sequence analysis of several species in the genus Tepidimonas and the description of a novel species *Tepidimonas charontis* sp. nov

**DOI:** 10.1099/ijsem.0.004563

**Published:** 2020-12-29

**Authors:** Luciana Albuquerque, Nadine Castelhano, Pedro Raposo, Hugo J. C. Froufe, Igor Tiago, Rita Severino, Inês Roxo, Inês Gregório, Cristina Barroso, Conceição Egas, Milton S. da Costa

**Affiliations:** ^1^​ Center for Neuroscience and Cell Biology, University of Coimbra, 3004-504 Coimbra, Portugal; ^2^​ Next Generation Sequencing Unit, Biocant, BiocantPark, Núcleo 04, Lote 8, 3060-197 Cantanhede, Portugal; ^3^​ Center for Functional Ecology, University of Coimbra, 3000-456, Coimbra, Portugal

In the published version of this article there were some errors in Table S1 which are corrected in this version.

The title of Table S1 should have read: ‘Differential characteristics of *Tepidimonas* species. 1, strain SPSP-6^T^; 2, strain SPSPC-18; 3, *Tepidimonas alkaliphilus* YIM 72238^T^; 4, *Tepidimonas aquatica* CLN-1^T^; 5*, Tepidimonas fonticaldi* AT-A2^T^; 6, *Tepidimonas ignava* SPS-1037^T^ *, 7, Tepidimonas sediminis* YIM 72259^T^; 8, *Tepidimonas taiwanensis* I1-1^T^; 9, *Tepidimonas thermarum* AA-1^T^. +, positive; −, negative; nd, not determined. Strains SPSP-6^T^, SPSPC-18, *Tepidimonas thermarum* AA-1^T^, *Tepidimonas ignava* SPS-1037^T^, *Tepidimonas aquatica* CLN-1^T^, *Tepidimonas taiwanensis* I1-1^T^ and *Tepidimonas fonticaldi* AT-A2^T^ assimilate succinate, l-glutamate and l-glutamine. Strains SPSP-6^T^, SPSPC-18, *Tepidimonas thermarum* AA-1^T^, *Tepidimonas ignava* SPS-1037^T^, *Tepidimonas aquatica* CLN-1^T^ and *Tepidimonas taiwanensis* I1-1^T^ assimilate lactate, pyruvate, acetate, but do not assimilate d-galactose, d-mannose, trehalose, cellobiose, melibiose, raffinose, d-ribose, d-xylose, d-arabinose, l-arabinose, l-rhamnose, l-fucose, l-sorbose, sucrose, lactose, maltose, ribitol, xylitol, sorbitol, erythritol, d-mannitol, *myo*-inositol, glycerol, benzoate, formate, glycine, l-methionine, l-serine and valine.’

On line 1 of the table ‘Characteristics, 8’ a reference letter was added in strain 8.

The table entries for rows ‘NaCl for growth (%), Optimum and Range’ column ‘8’ previously nd, should have read 0.2 and 0–1 respectively.

The table entry for row ‘L-lysine’ column ‘9’ previously − should have read +. The table entries for row ‘L-proline’ columns ‘4, 6 and 9’ previously, − should have read +and entries for columns ‘3, 5 and 7’ previously nd, should have read +.

The table entry for row ‘L-isoleucine’ column ‘5’ previously nd should have read -. The table entries for row ‘L-ornithine’ columns ‘4, 6 and 9’ previously −, should have read +.

The authors apologize for any inconvenience caused.

## Supplementary Data

Supplementary material 1

