# Supplementary material for: Comparative genome sequence analysis of several species in the genus Tepidimonas and the description of a novel species Tepidimonas charontis sp. nov
Source: Int J Syst Evol Microbiol. 2020 Jan 2;70(3):1596–604. doi: 10.1099/ijsem.0.003942 (PMC7386788; doi:10.1099/ijsem.0.003942)
Supplement: Supplementary material 1 [file ijsem-70-1596-s001.pdf]

Comparative genome sequence analysis of several species in the genus *Tepidimonas* and the description of a novel species *Tepidimonas charontis* sp. nov.

Luciana Albuquerque[1], Nadine Castelhano[1], Pedro Raposo[1], Hugo J. C. Froufe[2], Igor Tiago,[3] Rita Severino[1], Inês Roxo[1], Inês Gregório[1], Cristina Barroso[1,2], Conceição Egas[1,2] and Milton S. da Costa[1]*

[1]Center for Neuroscience and Cell Biology, University of Coimbra, 3004-504 Coimbra, Portugal

[2]Next Generation Sequencing Unit, Biocant, BiocantPark, Núcleo 04, Lote 8, 3060-197 Cantanhede, Portugal

[3]Center for Functional Ecology, University of Coimbra, 3000-456, Coimbra, Portugal

Correspondence: Milton S. da Costa, milton@ci.uc.pt

Keywords: Genome, New taxa, *Betaproteobacteria*, *Tepidimonas charontis* sp. nov.

Table S1. Differential characteristics of *Tepidimonas* species. 1, strain SPSP-6^T; 2, strain SPSPC-18; 3, *Tepidimonas alkaliphilus* YIM 72238 ^{T}; 4, *Tepidimonas aquatica* CLN-1^T; 5, *Tepidimonas fonticaldi* AT-A2^T; 6, *Tepidimonas ignava* SPS-1037^T, 7, *Tepidimonas sediminis* YIM 72259 ^{T}; 8, *Tepidimonas taiwanensis* I1-1^T; 9, *Tepidimonas thermarum* AA-1^T. +, positive; –, negative; nd, not determined. Strains SPSP-6^T, SPSPC-18, *Tepidimonas thermarum* AA-1^T, *Tepidimonas ignava* SPS-1037^T, *Tepidimonas aquatica* CLN-1^T, *Tepidimonas taiwanensis* I1-1^T and *Tepidimonas fonticaldi* AT-A2^T assimilate succinate, L-glutamate, L-glutamine, but do not assimilate glycerol. Strains SPSP-6^T, SPSPC-18, *Tepidimonas thermarum* AA-1^T, *Tepidimonas ignava* SPS-1037^T, *Tepidimonas aquatica* CLN-1^T and *Tepidimonas taiwanensis* I1-1^T assimilate lactate, pyruvate, acetate, but do not assimilate D-galactose, D-mannose, D-trehalose, D-cellobiose, D-melibiose, D-raffinose, D-ribose, D-xylose, D-arabinose, L-arabinose, L-rhamnose, L-fucose, L-sorbose, sucrose, lactose, maltose, ribitol, xylitol, sorbitol, erythritol, D-mannitol, *myo*-inositol, benzoate, formate, glycine, L-methionine, L-serine and valine.

**Characteristics**	**1***	**2***	**3**[a]	**4**[b,c]	**5**[a,d]	**6**[b.e]	**7**[a]	**8**[b,f]	**9**[b,d]
Temperature for growth (ºC)									
Optimum	50	50	45	50	55	50–55	45–50	55	50–55
Range	25–60	30–57.5	37–55	35–62	37–60	35–65	45–60	35–60	30–57.5
pH for growth									
Optimum	7.5–9.0	7.5–9.0	7.0–9.0	7.5–8.0	7.0	7.5–8.5	6.0–7.0	7.0	7.5–8.5
Range	6.5–9.5	6.5–9.5	6.0–11.0	6.5–9.5	7.0–9.0	6.5–9.5	6.0–9.0	6.0–8.0	6.0–9.5

NaCl for growth (%)									
Optimum	0	0	0.5	0	0.2	0	0.5	nd	0
Range	0–0.5	0–0.5	0–1	0–2	0–1	0–1	0–1	nd	0–1
Reduction of NO_3^- to NO_2^-	–	–	–	+	+	–	–	+	+[d] / –[b]
Assimilation of:									
D-glucose	–	–	–	–	–	–	–	+	–
D-fructose	–	–	–	–	–	–	–	+	–
α-ketoglutarate	–	–	nd	+	nd	+	nd	+	–
Malate	–	–	nd	+*/–[b]	–	+	nd	+	–
Citrate	–	–	nd	–	–	–	nd	+	–
Fumarate	–	–	nd	+	nd	+	nd	+	–
Aspartate	+	+	nd	+	–	+	nd	+	–
L-alanine	+	+	–	+	–	+	–	+	+
L-asparagine	+	+	+	+	–	+	–	+	+
L-histidine	–	–	–	–	+	–	+	+	–
L-lysine	+	–	+	–	+	–	+	+	–
L-proline	–	–	nd	–	nd	–	nd	+	–
L-arginine	–	–	–	–	+	–	+	+	–
L-isoleucine	+	+	nd	+	nd	+	nd	–	+
L-ornithine	+	–	nd	–	+	–	nd	+	–

L-threonine	–	–	+	–	–	–	–	–	–	
DNA G+C content (%)	66.6	nd	68.9	68.6	70.1	69.7	71.6	68.1	67.9	

*Data from this study.

[a]Data from [6].

[b]Data from [4].

[c]Data from [2].

[d]Data from [5].

[e]Data from [1].

[f]Data from [3].

Table S2. Genes involved in nitrate/nitrite metabolism in *Tepidimonas* genomes. 1, *Tepidimonas alkaliphilus* YIM 72238^{T} (VJNB00000000); 2, *Tepidimonas aquatica* CLN-1^{T} (VJNA00000000); 3, *Tepidimonas fonticaldi* AT-A2^{T} (VJOO00000000); 4, strain PL17 (GCF_001675355.1); 5, *Tepidimonas ignava* SPS-1037^{T} (VJNC00000000); 6, *Tepidimonas sediminis* YIM 72259 ^{T} (VJND00000000); 7, strain SPSP-6^{T} (VJON00000000); 8, *Tepidimonas taiwanensis* I1-1^{T} (VJOM00000000); 9, strain MB2 (GCF_001481285.1); 10, strain VT154-175 (GCF_000807215.1); 11, *Tepidimonas thermarum* AA-1^{T} (VJOL00000000).

	**1**	**2**	**3**	**4**	**5**	**6**	**7**	**8**	**9**	**10**	**11**
narGHIJ	nd	+	+	+	nd	nd	nd	+	nd	+	+
nirB	+	+	+	+	+	+	+	+	+	+	+
nirD	nd	+	+	+	nd	+	nd	nd	nd	nd	nd
nasA	nd	+	+	+	+	+	nd	+	nd	+	+
nirK	nd	+	+	+	nd	nd	nd	+	+	+	+
nirS	nd	nd	+	nd	nd	nd	nd	nd	nd	nd	nd
norB	nd	nd	+	nd	nd	nd	nd	nd	nd	nd	nd
norC	nd	nd	+	nd	nd	nd	nd	nd	nd	nd	nd

nd, not detected; *narGHIJ*, nitrate reductase complex; *nirB*, nitrite reductase (NADH dependent subunit); *nirD*, nitrite reductase subunit; *nasA*, nitrite reductase, large subunit; *nirK*, nitrite reductase, small subunit; *nirS*, nitrite reductase, monomer; *norB*, nitric oxide reductase, large subunit; *norC*, nitric oxide reductase, small subunit.

Table S3. Pairwise similarity values determined between the 16S rRNA sequence gene of strains SPSP-6^{T}, SPSPC-18^{T} and the type strains belonging to the genus *Tepidimonas*, and to species belonging to genera *Tepidicella* and *Acidovorax* of the family *Comamonadaceae*. 1, strain SPSP-6^{T} (MH590702); 2, strain SPSPC-18 (MH590703); 3, *Tepidimonas aquatica* CLN-1^{T} (AY324139); 4, *Tepidimonas ignava* SPS-1037^{T} (AF177943); 5, *Tepidimonas taiwanensis* I1-1^{T} (AY845054); 6, strain MB2 (NZ_LOQE01000009) (AUQ40_RS04785); 7, strain VT154-175 (NZ_JTKY01000044) (PL21 RS05080); 8, *Tepidimonas thermarum* AA1^{T} (AM042693); 9, *Tepidimonas alkaliphilus* YIM 72238^{T} (MF509266); 10, *Tepidimonas sediminis* YIM 72259^{T} (MF509190); 11, "*Tepidimonas arfidensis"* (AY594193); 12, strain PL17 (KF206381); 13, *Tepidimonas fonticaldi* AT-A2^{T} (JN713899); 14, *Tepidicella xavieri* TU-16^{T} (DQ295805); 15, *Acidovorax caeni* R-24608^{T} (AM084006).

	**1**	**2**	**3**	**4**	**5**	**6**	**7**	**8**	**9**	**10**	**11**	**12**	**13**	**14**	**15**
**1**	100.00														
**2**	100.00	100.00													
**3**	98.38	98.37	100.00												
**4**	98.07	97.94	97.98	100.00											
**5**	98.44	98.22	97.20	97.76	100.00										
**6**	98.44	98.29	97.31	97.85	99.93	100.00									
**7**	98.59	98.29	96.97	97.58	99.58	99.67	100.00								
**8**	96.88	96.73	95.76	97.04	96.71	96.96	97.29	100.00							
**9**	97.18	97.00	95.64	97.64	97.39	97.38	97.38	96.75	100.00						
**10**	97.25	97.14	96.25	97.63	97.10	97.09	97.02	96.60	98.62	100.00					
**11**	96.97	96.84	96.46	95.73	96.31	96.37	96.02	97.42	95.12	95.03	100.00				
**12**	97.20	97.03	96.78	95.86	96.43	96.53	96.19	97.55	95.25	95.17	99.65	100.00			
**13**	97.12	97.03	96.77	95.85	96.36	96.48	96.13	97.51	95.12	95.03	99.58	99.86	100.00		
**14**	94.42	94.38	94.66	95.47	94.61	94.73	94.59	95.61	94.09	94.14	94.82	9497	94.96	100.00	
**15**	93.23	93.24	93.27	93.28	93.35	92.82	92.49	94.57	93.37	93.35	95.25	95.44	95.37	94.12	100.00

Table S4. Average amino acid identity (AAI, %) values between genomes of members of the genus *Tepidimonas*, and strains *Acidovorax caeni* R-24608^{T} and *Tepidicella xavieri* TU-16^{T}. 1, *Tepidimonas alkaliphilus* YIM 72238^{T} (VJNB00000000); 2, *Tepidimonas aquatica* CLN-1^{T} (VJNA00000000); 3, *Tepidimonas fonticaldi* AT-A2^{T} (VJOO00000000); 4, strain PL17 (GCF_001675355.1); 5, *Tepidimonas ignava* SPS-1037^{T} (VJNC00000000); 6, *Tepidimonas sediminis* YIM 72259^{T} (VJND00000000); 7, strain SPSP-6^{T} (VJON00000000); 8, *Tepidimonas taiwanensis* I1-1^{T} (VJOM00000000); 9, strain MB2 (GCF_001481285.1); 10, strain VT154-175 (GCF_000807215.1); 11, *Tepidimonas thermarum* AA-1^{T} (VJOL00000000); 12, *Acidovorax caeni* R-24608^{T} (GCF_001298675.1); 13, *Tepidicella xavieri* TU-16^{T} (GCF_004363315.1).

	**1**	**2**	**3**	**4**	**5**	**6**	**7**	**8**	**9**	**10**	**11**	**12**	**13**
**1**	–	76.51	74.84	74.72	76.27	82.07	73.38	72.82	73.10	72.79	74.30	62.31	67.59
**2**	76.51	–	75.69	75.71	85.68	77.76	74.25	74.40	74.61	74.46	75.12	62.66	69.00
**3**	74.84	75.69	–	88.33	75.41	76.97	76.62	77.34	77.84	77.56	79.52	63.47	70.85
**4**	74.72	75.71	88.33	–	75.39	76.89	76.04	77.59	77.95	77.70	79.43	63.24	70.49
**5**	76.27	85.68	75.41	75.39	–	77.35	74.99	74.39	74.33	74.42	75.07	62.67	68.09
**6**	82.07	77.76	76.97	76.89	77.35	–	74.42	74.16	74.24	74.18	75.47	62.84	68.12
**7**	73.38	74.25	76.62	76.04	74.99	74.42	–	75.94	75.76	75.82	75.86	63.34	68.58
**8**	72.82	74.40	77.34	77.59	74.39	74.16	75.94	–	88.66	89.96	77.52	63.16	69.21
**9**	73.10	74.61	77.84	77.95	74.33	74.24	75.76	88.66	–	88.31	77.48	63.15	69.89
**10**	72.79	74.46	77.56	77.70	74.42	74.18	75.82	89.96	88.31	–	77.55	63.16	69.38
**11**	74.30	75.12	79.52	79.43	75.07	75.47	75.86	77.52	77.48	77.55	–	63.26	69.72
**12**	62.31	62.66	63.47	63.24	62.67	62.84	63.34	63.16	63.15	63.16	63.26	–	64.35
**13**	67.59	69.00	70.85	70.49	68.09	68.12	68.58	69.21	69.89	69.38	69.72	64.35	–

Table S5. Digital DNA-DNA hybridization (dDDH, %) values between genomes of members of the species genus *Tepidimonas*, and strains *Acidovorax caeni* R-24608^T and *Tepidicella xavieri* TU-16^T. 1, *Tepidimonas alkaliphilus* YIM 72238^T (VJNB00000000); 2, *Tepidimonas aquatica* CLN-1^T (VJNA00000000); 3, *Tepidimonas fonticaldi* AT-A2^T (VJOO00000000); 4, strain PL17 (GCF_001675355.1); 5, *Tepidimonas ignava* SPS-1037^T (VJNC00000000); 6, *Tepidimonas sediminis* YIM 72259^T (VJND00000000); 7, strain SPSP-6^T (VJON00000000); 8, *Tepidimonas taiwanensis* I1-1^T (VJOM00000000); 9, strain MB2 (GCF_001481285.1); 10, strain VT154-175 (GCF_000807215.1); 11, *Tepidimonas thermarum* AA-1^T (VJOL00000000); 12, *Acidovorax caeni* R-24608^T (GCF_001298675.1); 13, *Tepidicella xavieri* TU-16^T (GCF_004363315.1).

	**1**	**2**	**3**	**4**	**5**	**6**	**7**	**8**	**9**	**10**	**11**	**12**	**13**
**1**	–	24.2	25.1	24.9	23.9	34.8	22.4	23.1	23.3	23.2	22.9	19.2	20.1
**2**	24.2	–	25.1	24.5	53.0	25.4	22.9	23.1	23.8	23.2	22.9	19.3	21.1
**3**	25.1	25.1	–	80.1	23.2	26.2	25.1	25.5	26.0	25.5	26.1	20.4	21.7
**4**	24.9	24.5	80.1	–	22.9	26.0	23.9	25.2	25.6	25.2	26.2	19.8	21.2
**5**	23.9	53.0	23.2	22.9	–	25.1	24.2	23.4	23.6	23.2	22.8	19.3	20.6
**6**	34.8	25.4	26.2	26.0	25.1	–	22.9	24.0	24.1	24.0	23.6	19.4	20.2
**7**	22.4	22.9	25.1	23.9	24.2	22.9	–	23.8	23.8	23.7	23.2	19.2	21.3
**8**	23.1	23.1	25.5	25.2	23.4	24.0	23.8	–	79.8	87.2	24.8	19.8	21.0
**9**	23.3	23.8	26.0	25.6	23.6	24.1	23.8	79.8	–	76.0	24.8	19.8	21.8
**10**	23.2	23.2	25.5	25.2	23.2	24.0	23.7	87.2	76.0	–	24.8	19.8	21.2
**11**	22.9	22.9	26.1	26.2	22.8	23.6	23.2	24.8	24.8	24.8	–	19.7	20.4
**12**	19.2	19.3	20.4	19.8	19.3	19.4	19.2	19.8	19.8	19.8	19.7	–	20.4
**13**	20.1	21.1	21.7	21.2	20.6	20.2	21.3	21.0	21.8	21.2	20.4	20.4	–

Table S6. Fatty acid composition of species of the genus *Tepidimonas* grown on R2A medium at 50 ºC for 24 h. 1, strain SPSP-6^T; 2, strain SPSPC-18; 3, *Tepidimonas alkaliphilus* YIM 72238 ^{T}; 4, *Tepidimonas aquatica* CLN-1^T; *5, Tepidimonas fonticaldi* AT-A2^T*;* 6, *Tepidimonas ignava* SPS-1037^T*,* 7, *Tepidimonas sediminis* YIM 72259 ^{T}; 8, *Tepidimonas taiwanensis* I1-1^T*;* 9, *Tepidimonas thermarum* AA-1^T.

**Fatty acids**	**ECL**	**1**	**2**	**3**	**4**	**5**	**6**	**7**	**8**	**9**
$C_{8:0}$ 3-OH	9.392	3.5 ± 0.3	2.6 ± 0.4	2.6 ± 0.2	2.5 ± 0.6	4.2 ± 0.6	3.2 ± 0.6	2.8 ± 0.1	3.5 ± 0.5	3.5 ± 0.6
$C_{9:0}$ 3-OH	10.408	–	–	–	–	–	tr	–	–	–
$C_{15:1}$ ω6c	14.856	tr	–	tr	tr	tr	2.5 ± 0.5	0.8 ± 0.1	–	1.0 ± 0.1
$C_{15:0}$	15.000	0.8 ± 0.1	tr	1.8 ± 0.1	0.5 ± 0.1	1.4 ± 0.1	6.6 ± 0.7	6.3 ± 0.3	0.8 ± 0.1	3.6 ± 0.1
$C_{16:0}$ iso	15.627	–	–	–	–	0.8 ± 0.1	–	–	–	–
Summed feature 3	15.822	24.8 ± 0.4	15.7 ± 0.7	13.5 ± 0.3	12.3 ± 0.8	15.2 ± 1.0	20.4 ± 1.9	9.0 ± 0.4	14.5 ± 0.6	16.7 ± 0.8
$C_{16:0}$	16.000	45.4 ± 1.0	50.6 ± 0.8	39.9 ± 0.9	45.9 ± 1.5	38.2 ± 1.0	29.3 ± 1.0	30.1 ± 0.9	45.8 ± 1.2	40.9 ± 1.1
Unknown 16.090	16.090	–	–	–	1.4 ± 0.3	0.6 ± 0.3		–		
$C_{17:1}$ ω8c	16.792	–	–	0.6 ± 0.1	–	–	0.9 ± 0.1	0.8 ± 0.1	–	–
$C_{17:1}$ ω6c	16.860	–	–	–	–	–	–	–	–	–
$C_{17:0}$ cyclo	16.888	6.1 ± 0.4	12.4 ± 0.4	3.5 ± 0.1	25.3 ± 2.0	17.5 ± 0.8	6.7 ± 0.4	7.3 ± 0.4	11.5 ± 0.4	12.6 ± 0.5
$C_{17:0}$	17.000	1.9 ± 0.2	0.6 ± 0.1	8.5 ± 0.4	4.6 ± 0.6	4.9 ± 0.6	14.9 ± 2.8	15.9 ± 0.5	2.8 ± 0.2	5.8 ± 0.4
Unknown 17.747	17.747	–	–	–	1.0 ± 0.3	0.6 ± 0.1	–	–	–	–
Summed feature 8	17.823	11.9 ± 0.5	11.2 ± 0.7	17.5 ± 0.5	–	10.7 ± 0.3	7.6 ± 1.0	11.9 ± 0.4	12.8 ± 0.3	9.3 ± 0.5
$C_{18:0}$	18.000	2.3 ± 0.3	1.9 ± 0.2	4.9 ± 0.3	4.4 ± 0.8	2.1 ± 0.4	1.6 ± 0.5	4.6 ± 0.3	3.0 ± 0.3	1.8 ± 0.4
$C_{18:1}$ ω7c 11-methyl	18.081	–	0.7 ± 0.1	4.1 ± 0.2	–	–	1.2 ± 0.4	2.4 ± 0.2	1.9 ± 0.3	2.0 ± 0.3
$C_{18:0}$ 12-methyl	18.430	tr	0.7 ± 0.1	–	1.6 ± 0.2	1.0 ± 0.1	–	–	0.7 ± 0.1	0.7 ± 0.1
$C_{19:0}$ iso	18.639	–	–	–	0.8 ± 0.2	tr	–	–	–	–

Unknown 18.814	18.814	–	2.1 ± 0.1	–	–	–	–	–	–	–
Summed feature 7	18.846	2.2 ± 0.2	–	1.9 ± 0.1	–	–	3.6 ± 0.6	4.7 ± 0.2	0.7 ± 0.1	tr
$C_{19:0}$ cyclo *ω*8c	18.902	tr	1.0 ± 0.1	1.0 ± 0.1	–	1.4 ± 0.2	tr	1.9 ± 0.1	1.0 ± 0.1	–

Results are percentage of the total fatty acids. ±, results are the mean plus the standard deviation of two to four analyses of the two strains; values for fatty acids present at less than 0.5% in all strains are not shown; tr, trace (< 0.5%); –, not detected. ECL, equivalent chain length. A summed feature represents groups of two or three fatty acids that could not be separated by GLC with the MIDI System: summed feature 3 comprises $C_{16:1}$ ω7*c* and/or $C_{16:1}$ ω6*c* and/or iso-$C_{15:0}$ 2-OH; summed feature 8 comprises $C_{18:1}$ ω7*c* and/or $C_{18:1}$ ω6*c*; summed feature 7 comprises unknown 18.846 and/or $C_{19:1}$ ω6*c*.

Table S7. Fatty acid composition of species of the genus *Tepidimonas* grown on Degryse medium 162 agar plates at 50 ºC for 24 h. 1, strain SPSP-6^{T}; 2, strain SPSPC-18; 3, *Tepidimonas alkaliphilus* YIM 72238; 4, *Tepidimonas aquatica* CLN-1^{T}; *5, Tepidimonas fonticaldi* AT-A2^{T}*;* 6, *Tepidimonas ignava* SPS-1037^{T}; 7*, Tepidimonas sediminis* YIM 72259^{T}; 8, *Tepidimonas taiwanensis* I1-1^{T}*;* 9, *Tepidimonas thermarum* AA-1^{T}.

**Fatty acids**	**ECL**	**1**	**2**	**3**	**4**	**5**	**6**	**7**	**8**	**9**
$C_{8:0}$ 3-OH	9.392	3.2 ± 0.7	2.1 ± 0.1	3.3 ± 0.4	3.2 ± 0.3	3.8 ± 0.3	2.5 ± 0.6	3.1 ± 0.1	3.7 ± 0.5	2.4 ± 0.5
$C_{15:1}$ ω6c	14.856	0.7 ± 0.1	tr	0.6 ± 0.1	tr	tr	7.0 ± 0.6	2.6 ± 0.3	0.7 ± 0.1	2.4 ± 0.5
$C_{15:0}$	15.000	1.5 ± 0.2	0.5 ± 0.2	1.6 ± 0.2	0.7 ± 0.1	0.7 ± 0.1	9.0 ± 0.7	8.3 ± 0.5	1.0 ± 0.3	2.9 ± 0.3
Summed feature 3	15.822	30.8 ± 2.2	27.7 ± 4.1	21.2 ± 1.2	26.7 ± 1.9	17.5 ± 1.4	29.6 ± 1.2	15.5 ± 0.4	31.8 ± 1.5	37.1 ± 0.9
$C_{16:0}$	16.000	39.1 ± 2.1	44.5 ± 5.1	39.9 ± 2.0	37.5 ± 1.2	38.6 ± 1.3	19.2 ± 0.6	25.4 ± 0.9	38.7 ± 1.2	35.3 ± 1.1
$C_{17:1}$ ω8c	16.792	–	–	–	–	–	1.1 ± 0.1	1.1 ± 0.1	–	–
$C_{17:1}$ ω6c	16.860	–	–	–	–	–	3.8 ± 0.2	4.8 ± 0.2	–	–
$C_{17:0}$ cyclo	16.888	2.7 ± 0.2	4.5 ± 2.0	2.2 ± 0.1	22.0 ± 3.6	13.8 ± 0.5	–	–	5.8 ± 0.5	6.1 ± 0.7
$C_{17:0}$	17.000	2.9 ± 0.2	1.2 ± 0.1	6.8 ± 0.4	2.4 ± 0.2	3.9 ± 0.3	16.5 ± 1.2	17.7 ± 0.5	2.2 ± 0.2	3.5 ± 0.4
Summed feature 8	17.823	8.7 ± 0.6	12.2 ± 0.9	15.0 ± 0.7	–	14.0 ± 0.7	6.2 ± 0.5	11.0 ± 0.6	13.0 ± 0.7	6.8 ± 0.5
$C_{18:0}$	18.000	2.3 ± 0.3	1.7 ± 0.9	3.2 ± 0.3	2.0 ± 0.2	2.8 ± 0.2	0.7 ± 0.1	3.1 ± 0.1	1.2 ± 0.2	0.8 ± 0.1
$C_{18:1}$ ω7c 11-methyl	18.081	tr	1.0 ± 0.3	4.0 ± 0.3	–	–	2.0 ± 0.2	2.2 ± 0.1	0.7 ± 0.1	1.0 ± 0.2
$C_{18:0}$ 12-methyl	18.430	tr	–	–	1.4 ± 0.3	0.9 ± 0.1	–	–	0.5 ± 0.1	tr
Summed feature 7	18.846	1.4 ± 0.2	1.5 ± 0.2	1.0 ± 0.1	–	–	tr	3.4 ± 0.2	–	–
$C_{19:0}$ cyclo ω8c	18.902	–	tr	tr	–	1.7 ± 0.2	–	tr	tr	–

Results are percentage of the total fatty acids. ±, results are the mean plus the standard deviation of two to four analyses of the two strains; values for fatty acids present at less than 0.5% in all strains are not shown; tr, trace (< 0.5%); –, not detected. ECL, equivalent chain length. A summed feature represents groups of two or three fatty acids that could not be separated by GLC with the MIDI System: summed feature 3 comprises $C_{16:1}$ ω7*c* and/or $C_{16:1}$ ω6*c* and/or iso-$C_{15:0}$ 2-OH; summed feature 8 comprises $C_{18:1}$ ω7*c* and/or $C_{18:1}$ ω6*c*; summed feature 7 comprises unknown 18.846 and/or $C_{19:1}$ ω6*c*.

Figure legends

Figure S1. Phylogenetic reconstruction based on 16S rRNA genes of *Tepidimonas* spp. using the maximum likelihood algorithm. The numbers at branching points represent bootstrap values from 1000 replications. Bar, 0.02 substitutions per nucleotide position. The tree was rooted using the sequence of *Alcaligenes faecalis* ATCC 8750^{T} (D88008).

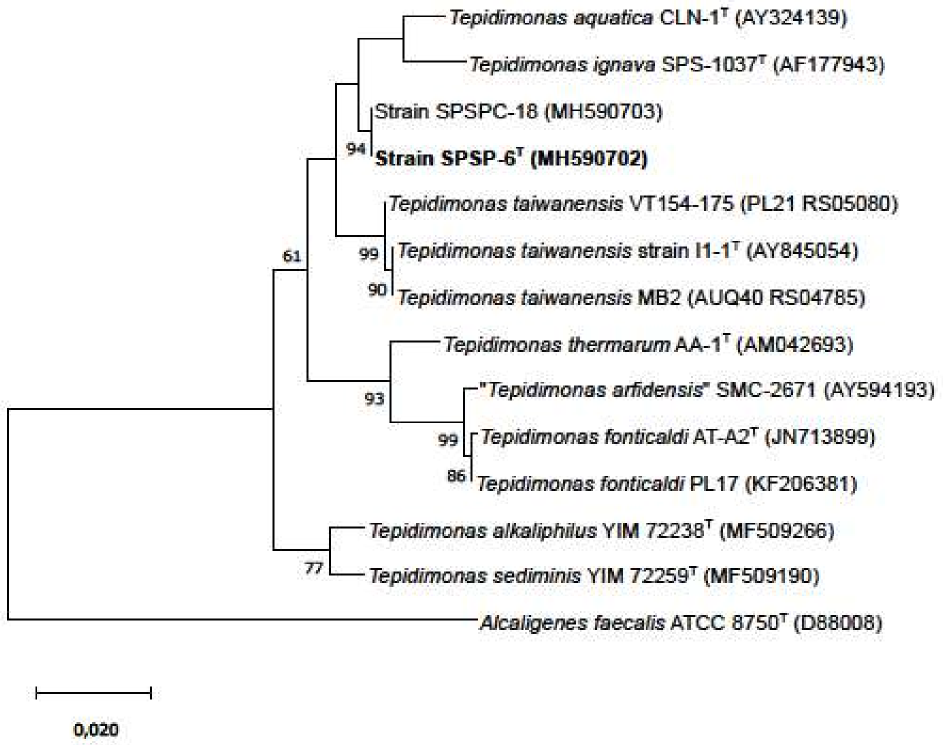
Tepidimonas aquatica CLN-1T (AY324139)
Tepidimonas ignava SPS-1037T (AF177943)
Strain SPSPC-18 (MH590703)
94
Strain SPSP-6T (MH590702)
Tepidimonas taiwanensis VT154-175 (PL21 RS05080)
99
Tepidimonas taiwanensis strain I1-1T (AY845054)
61
90
Tepidimonas taiwanensis MB2 (AUQ40 RS04785)
Tepidimonas thermarum AA-1T (AM042693)
"Tepidimonas arfidensis" SMC-2671 (AY594193)
93
99
Tepidimonas fonticaldi AT-A2T (JN713899)
86
Tepidimonas fonticaldi PL17 (KF206381)
Tepidimonas alkaliphilus YIM 72238T (MF509266)
77
Tepidimonas sediminis YIM 72259T (MF509190)
Alcaligenes faecalis ATCC 8750T (D88008)
0,020

Figure S1.
