# Supplementary material for: Corrigendum: Comparative genome sequence analysis of several species in the genus Tepidimonas and the description of a novel species Tepidimonas charontis sp. nov
Source: Int J Syst Evol Microbiol. 2020 Dec 29;70(12):6539. doi: 10.1099/ijsem.0.004563 (PMC8049489; doi:10.1099/ijsem.0.004563)
Supplement: Supplementary material 1 [file ijsem-70-6539-s001.pdf]

Table S1. Differential characteristics of *Tepidimonas* species. 1, strain SPSP-6^T; 2, strain SPSPC-18; 3, *Tepidimonas alkaliphilus* YIM 72238^T; 4, *Tepidimonas aquatica* CLN-1^T; 5*, Tepidimonas fonticaldi* AT-A2^T*;* 6, *Tepidimonas ignava* SPS-1037^T*, 7, Tepidimonas sediminis* YIM 72259^T; 8, *Tepidimonas taiwanensis* I1-1^T*;* 9, *Tepidimonas thermarum* AA-1^T. +, positive; –, negative; nd, not determined. Strains SPSP-6^T, SPSPC-18, *Tepidimonas thermarum* AA-1^T, *Tepidimonas ignava* SPS-1037^T, *Tepidimonas aquatica* CLN-1^T, *Tepidimonas taiwanensis* I1-1^T and *Tepidimonas fonticaldi* AT-A2^T assimilate succinate, L-glutamate and L-glutamine. Strains SPSP-6^T, SPSPC-18, *Tepidimonas thermarum* AA-1^T, *Tepidimonas ignava* SPS-1037^T, *Tepidimonas aquatica* CLN-1^T and *Tepidimonas taiwanensis* I1-1^T assimilate lactate, pyruvate, acetate, but do not assimilate D-galactose, D-mannose, D-trehalose, D-cellobiose, D-melibiose, D-raffinose, D-ribose, D-xylose, D-arabinose, L-arabinose, L-rhamnose, L-fucose, L-sorbose, sucrose, lactose, maltose, ribitol, xylitol, sorbitol, erythritol, D-mannitol, *myo*-inositol, glycerol, benzoate, formate, glycine, L-methionine, L-serine and valine.

**Characteristics**	**1**[*]	**2**[*]	**3**[a]	**4**[b,c]	**5**[a,d]	**6**[b.e]	**7**[a]	**8**[b,d,f]	**9**[b,d]
Temperature for growth (ºC)									
Optimum	50	50	45	50	55	50–55	45–50	55	50–55
Range	25–60	30–57.5	37–55	35–62	37–60	35–65	45–60	35–60	30–57.5
pH for growth									
Optimum	7.5–9.0	7.5–9.0	7.0–9.0	7.5–8.0	7.0	7.5–8.5	6.0–7.0	7.0	7.5–8.5
Range	6.5–9.5	6.5–9.5	6.0–11.0	6.5–9.5	7.0–9.0	6.5–9.5	6.0–9.0	6.0–8.0	6.0–9.5
NaCl for growth (%)									
Optimum	0	0	0.5	0	0.2	0	0.5	0.2	0
Range	0–0.5	0–0.5	0–1	0–2	0–1	0–1	0–1	0–1	0–1

Reduction of NO_3^- to NO_2^-	–	–	–	+	+	–	–	+	+[d]/–[b]
Assimilation of:									
D-glucose	–	–	–	–	–	–	–	+	–
D-fructose	–	–	–	–	–	–	–	+	–
α-ketoglutarate	–	–	nd	+	nd	+	nd	+	–
Malate	–	–	nd	+[*]/–[b]	–	+	nd	+	–
Citrate	–	–	nd	–	–	–	nd	+	–
Fumarate	–	–	nd	+	nd	+	nd	+	–
Aspartate	+	+	nd	+	–	+	nd	+	–
L-alanine	+	+	–	+	–	+	–	+	+
L-asparagine	+	+	+	+	–	+	–	+	+
L-histidine	–	–	–	–	+	–	+	+	–
L-lysine	+	–	+	–	+	–	+	+	+
L-proline	–	–	+	+	+	+	+	+	+
L-arginine	–	–	–	–	+	–	+	+	–
L-isoleucine	+	+	nd	+	–	+	nd	–	+
L-ornithine	+	–	nd	+	+	+	nd	+	+
L-threonine	–	–	+	–	–	–	–	–	–
DNA G+C content (%)	66.6	nd	68.9	68.6	70.1	69.7	71.6	68.1	67.9

[*]Data from this study.

[a]Data from [6].

[b]Data from [4].

[c]Data from [2].

[d]Data from [5].

[e]Data from [1].

[f]Data from [3].
